# Spontaneous rupture of isolated splenic hydatid cyst without acute abdomen: A case report

**DOI:** 10.1002/ccr3.2424

**Published:** 2019-09-26

**Authors:** Suman Acharya, Bikal Ghimire, Nischal Khanal

**Affiliations:** ^1^ Tribhuwan University Teaching Hospital Maharajgunj Nepal

**Keywords:** rupture, splenectomy, splenic hydatid cyst

## Abstract

Isolated splenic hydatid cyst is a very rare manifestation of hydatid cyst. Rarely, patients present late after the cyst is ruptured. They might not present with usual presentations such as the acute abdomen, anaphylaxis, or urticarial rashes but only with a distended abdomen. Splenectomy is the emergency operative management.

## Graphical abstract

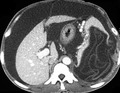

## 1 INTRODUCTION

Splenic hydatid cyst is a very rare extrahepatic manifestation of hydatid cyst. Once ruptured into peritoneal cavity, splenic hydatid cyst presents with features of acute abdomen, abdominal distension, urticarial rashes, or anaphylactic shock. We report a case of middle‐aged woman with ruptured splenic hydatid cyst who presented with distended abdomen.

Hydatid disease is a zoonotic disease, common in Mediterranean region caused by Echinococcus granulosus and less frequently by Echinococcus multilocularis. Devleesschauwer et al reported echinococcosis is probably endemic and quantifiable in Nepal with DALYs of 667.^1^ It is acquired after accidental ingestion of eggs shed in the feces of definitive hosts, dogs and foxes. It develops as a cystic lesion in the human organs which is termed cystic echinococcosis (CE). Cyst consists of outer layer pericyst, ectocyst, and endocyst. Liver is the most common site for cyst representing 70% of all hydatid cysts followed by lungs (20%‐25%).^2^, ^3^, ^4^, ^5^ Hammaci et al and Col et al reported incidence rates of 16.3% and 8%, respectively, in unusual locations like spleen, kidney, peritoneum, retroperitoneum, bone, thyroid, heart, and brain.^2^, ^3^

## 2 CASE

A 49‐year‐old Hindu woman presented to Tribhuwan University Teaching Hospital emergency with abdominal pain for 3 months and generalized abdominal distension for 10 days. Abdominal pain, intermittent in nature was localized on left upper quadrant with intensity of 4/10 in the numeric rating scale of pain. Her abdominal pain worsened abruptly 10 days back and had multiple episodes of vomiting and diarrhea on the same day followed by brief loss of consciousness and dizziness. She also complained of decreased urine output for 10 days following the episode along with gradual and progressive bilateral limb swelling. She had no fever, melena, yellowish discoloration of body, or history of abdominal trauma. She was an ex‐smoker and was regularly taking medications for COPD. She had no other comorbidities. She had not worked directly in contact with sheep or dogs before.

On presentation at the ER, she was ill looking with bilateral lower limb edema. Her blood pressure was 90/60 mm Hg, afebrile with pulse rate of 96 bpm and normal respiratory rate. There were no rashes in her abdomen which was soft, distended, and nontender. On percussion, there were dull notes over the abdomen with shifting dullness. Spleen was palpable; however, liver was not enlarged. Chest and CVS examinations were unremarkable.

**Figure 1:**
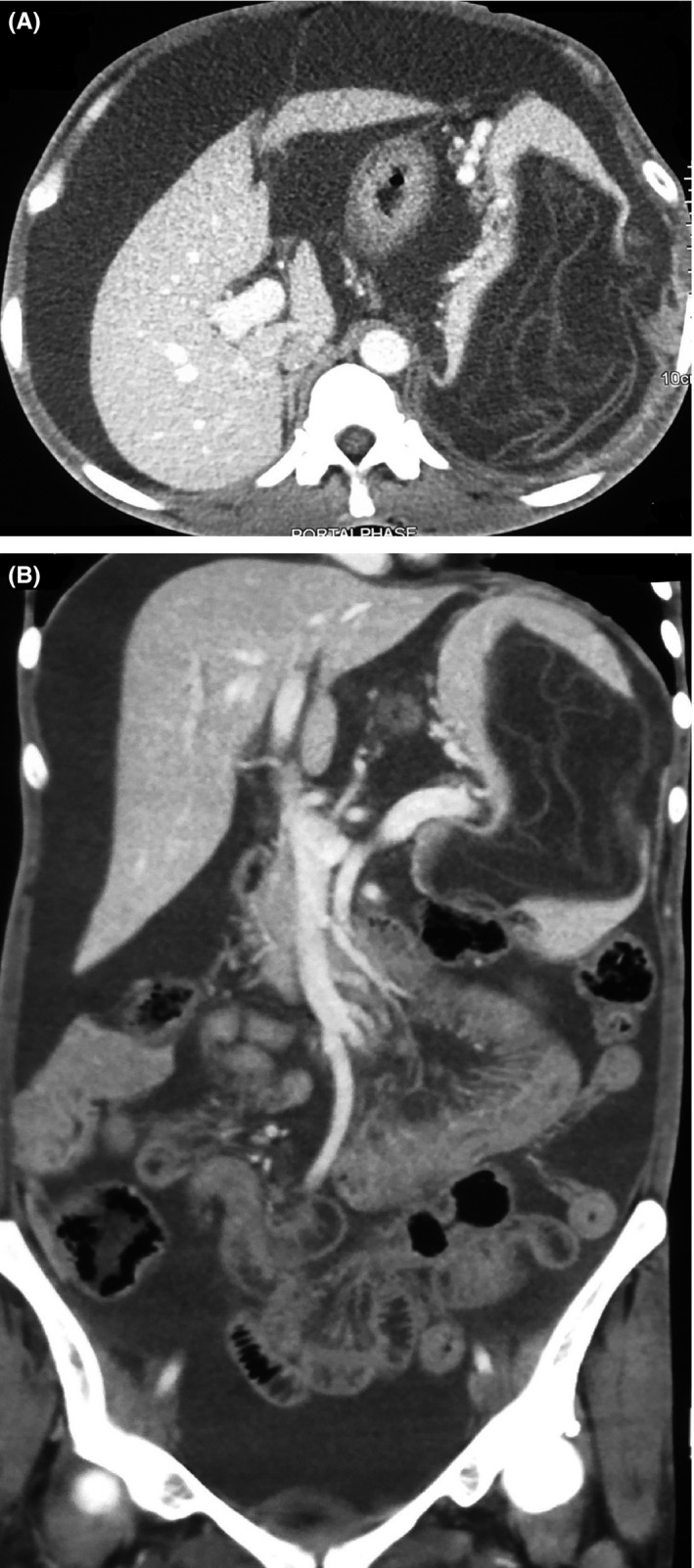
CT finding suggesting gross ascites and nonenhancing hypodense cystic lesion within splenic parenchyma with floating membrane within; feature suggestive of ruptured hydatid cyst (A,B)

Hematology revealed total white blood cell count of 4700/cc with 40% eosinophils. Hemoglobin was normal (12.4 gm/dL) along with normal coagulation essay. Biochemistry revealed normal renal function and normal liver function tests except hypoalbuminemia (24 g/L). Abdominal sonogram revealed gross free fluid in abdomen and pelvis. Ascitic fluid assay revealed white blood cell count of 1400/cc with 40% polymorph with negative ADA and low SAAG (9.5 g/L). Routine examination of stool and urine were unremarkable. CECT revealed splenomegaly with nonenhancing cystic lesion of size 14.5 × 11.5 × 10.6 cm within splenic parenchyma extending into perisplenic region with floating membrane within the cyst. (Figure 1A,B) Hepatomegaly with gross ascites, portal hypertension, and multiple collaterals in splenic hilum and perigastric region were also noted.

The provisional diagnosis of ascites with presinusoidal portal hypertension secondary to ruptured splenic hydatid cyst was made. She was resuscitated for suspected anaphylaxis with inj. hydrocortisone and inj. Chlorpheniramine and IV fluids in ER. Oral Albendazole 400 mg twice a day was started. She was then shifted to the operating room for an emergency laparotomy and splenectomy.

**Figure 2:**
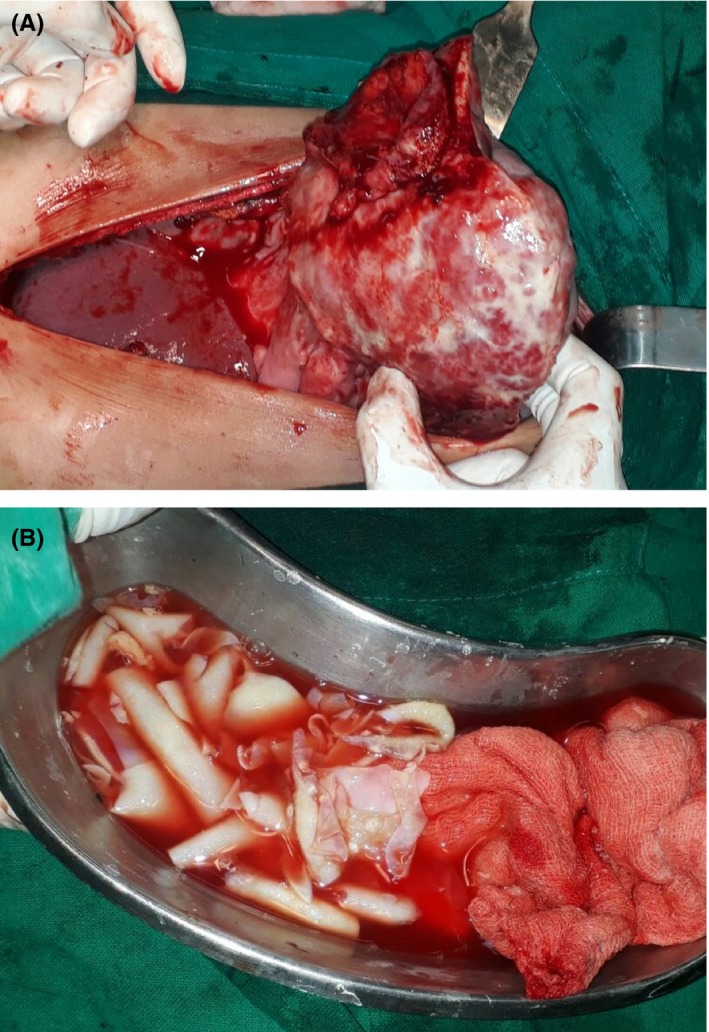
Intraoperative images of ruptured spleen along with cyst membranes (A,B)

Intra‐operatively, dense adhesions over the stomach, splenic flexure, transverse colon, diaphragm, and omentum were noted with ruptured hydatid cyst of spleen. (Figure 2A,B) Four liters of ascitic fluid was removed and peritoneal cavity was thoroughly washed with normal saline. Histopathological examination of splenic and membranous tissue confirmed the diagnosis later. Postoperative course was uneventful and she was vaccinated for pneumococcus, meningococcus and Haemophilus influenzae type B (Hib). She was discharged with oral albendazole for 3 months. Three months later, there were no evidences of complications or recurrences.

## 3 DISCUSSION

Liver is the most common site for cystic echinococcosis with incidence ranging from 50% to 75% of all hydatid cyst followed by lungs (5%‐25%).^2^, ^3^, ^4^, ^6^ The hexacanth embryo of Echinococcus is usually trapped in the liver (first Lemman's filter) and/or lung (second Lemman's filter) but can disseminate to any organs once it enters systemic circulation.^4^ Spleen is rarely involved even in endemic regions with incidence of 1%‐5%.^4^ Splenic hydatid cyst usually grow very slowly and can be asymptomatic for years or can present with only left sided mild abdominal discomfort.^2^, ^4^, ^7^

Hydatid cysts rupture either after trauma or spontaneously due to raised intracystic pressure. Beyrouti et al reported that only 1.7% of the total studied cases of hydatid cysts presented acutely with rupture in the peritoneal cavity.^8^ Once ruptured into peritoneal cavity, the patient can present with acute abdomen, distension, urticarial rashes and anaphylactic shock.^6^, ^9^ The case presented here did not have acute abdomen or urticarial rashes but had abdominal distension. Eosinophilia was present in this case which is reported only in 25% of hydatid cysts.^3^ USG is the gold standard diagnostic tool and is diagnostic in 95% cases. CT scan has superior diagnostic sensitivity and specificity.^10^, ^11^

Emergency splenectomy is the treatment of choice in case of ruptured splenic cysts.^2^, ^4^, ^12^ However, few studies have suggested higher morbidity and mortality due to overwhelming postsplenectomy infections (OPSIs) among total splenectomy patients advocating for partial splenectomy whenever possible. Atmazidis et.al demonstrated that there was no higher risk of recurrence of hydatid cyst in patients undergoing spleen preserving surgery when compared to splenectomy.^4^, ^7^, ^12^ Peritoneal washing with scolicidal agents like hypertonic saline, povidone iodine, hydrogen peroxide may be effective in killing the scolices although they can be damaging to peritoneal surface. Therefore, normal saline is recommended for lavage.^13^ Oral Albendazole for 3 months following surgery is recommended to prevent recurrence of hydatid cysts.^6^ Meticulous follow‐up is essential to timely diagnose and manage recurrence of hydatid cyst. In cases of ruptured cystic echinococcosis into peritoneum, recurrence rates vary with 6.7%, 14%, and 28% recurrence rates reported by Beyrouti et.al, Derici et al and Kurt et.al, respectively.^8^, ^13^, ^14^

## 4 CONCLUSION

Ruptured splenic hydatid cyst, a very rare entity presents usually with acute abdomen, anaphylaxis but it can present with distended abdomen only. US/CT can be diagnostic and Emergency laparotomy and splenectomy should be done. Postsurgical albendazole therapy for 3 months is advised to prevent recurrence along with meticulous follow‐up.

## CONFLICT OF INTEREST

None declared.

## AUTHORS CONTRIBUTION

SA wrote the manuscript while BG and NK were involved in editing. All three authors were involved in management of patient.

## ETHICAL APPROVAL

Not applicable.

## CONSENT

Patient provided informed consent for publications.
